# Metaviromics Reveals Unknown Viral Diversity in the Biting Midge *Culicoides impunctatus*

**DOI:** 10.3390/v11090865

**Published:** 2019-09-17

**Authors:** Sejal Modha, Joseph Hughes, Giovanni Bianco, Heather M. Ferguson, Barbara Helm, Lily Tong, Gavin S. Wilkie, Alain Kohl, Esther Schnettler

**Affiliations:** 1 MRC-University of Glasgow Centre for Virus Research, Glasgow G61 1QH, UK; 2 Institute of Biodiversity, Animal Health and Comparative Medicine, University of Glasgow, Glasgow G12 8QQ, UK

**Keywords:** metaviromics, RNA viruses, *Culicoides impunctatus*

## Abstract

Biting midges (*Culicoides* species) are vectors of arboviruses and were responsible for the emergence and spread of *Schmallenberg virus* (SBV) in Europe in 2011 and are likely to be involved in the emergence of other arboviruses in Europe. Improved surveillance and better understanding of risks require a better understanding of the circulating viral diversity in these biting insects. In this study, we expand the sequence space of RNA viruses by identifying a number of novel RNA viruses from *Culicoides impunctatus* (biting midge) using a meta-transcriptomic approach. A novel metaviromic pipeline called MetaViC was developed specifically to identify novel virus sequence signatures from high throughput sequencing (HTS) datasets in the absence of a known host genome. MetaViC is a protein centric pipeline that looks for specific protein signatures in the reads and contigs generated as part of the pipeline. Several novel viruses, including an alphanodavirus with both segments, a novel relative of the Hubei sobemo-like virus 49, two rhabdo-like viruses and a chuvirus, were identified in the Scottish midge samples. The newly identified viruses were found to be phylogenetically distinct to those previous known. These findings expand our current knowledge of viral diversity in arthropods and especially in these understudied disease vectors.

## 1. Introduction

Viruses are widespread and are present in all forms of living organisms where they use their hosts to multiply and spread [1,2,3]. Advances in high throughput sequencing (HTS) technologies continue to expand the known diversity of RNA viruses. Indeed, the use of HTS technologies using meta-transcriptomic and metagenomic approaches has enabled the identification of a wide range of novel viruses in all organisms and this range is shown to be particularly broad for arthropods [4,5]. Viral metagenomics has been widely presented and accepted as one of the most unbiased methods for the characterisation of viral sequences [6,7] and, the International Committee on Taxonomy of Viruses (ICTV) now incorporates viruses known from metagenomic data into the official taxonomic scheme to better understand global virus diversity [8].

Arthropods have been shown to be major vectors of viruses [9,10,11,12]. However, there are still gaps in our knowledge of the vector virome outside of the human and animal pathogens they transmit to. Shi et al [5] identified 1445 RNA viruses from a metaviromics analysis of 220 invertebrate species. Furthermore, a number of the newly identified RNA viruses were sufficiently divergent from already known species to be categorised as novel species [5]. A similar study focusing on arthropods led to the discovery of 112 novel viruses from 70 distinct arthropod species and hypothesised that arthropods must have played a major role in shaping virus diversity and evolution [4]. A number of viruses identified in the latter study have been shown to be ancestors of disease-causing viruses such as influenza and the filoviruses. Additionally, variations in the genomic arrangements of these viruses suggest that arthropods have played an important role in the evolution of segmentation [13]. In addition to these large studies carried out in samples collected in China, a number of other studies have shed light on the diversity of viruses that exist in distinct arthropod species in other geographic regions.

Metaviromic studies have often focused on arthropod species of economic importance such as the honeybee or vectors. For example, seven novel viruses were discovered in geographically distinct honey bee populations [14]. In vectors, a high-resolution metatranscriptomic approach identified 19 new viruses within the *Aedes camptorhynchus*, *Ae. alboannulatus*, *Culex globocoxitus*, *Cx. australicus*, *Cx. quinquefasciatus*, *Cx. Globocoxitus*, and *Cx. australicus* populations in Western Australia [15]. The latter study found that viral diversity and abundance within the *Culex* genus were higher compared to the virome of the *Aedes* genus [15]. Additionally, metagenomic approaches led to the identification of four novel viruses in Australian mosquitoes: Two rhabdoviruses namely North Creek Virus, isolated from *Culex sitiens*, and Beaumont Virus, isolated from *Anopheles annulipes*; and two bunyaviruses, Murrumbidgee virus and Salt Ash Virus, isolated from *An. annulipes* mosquitoes [16]. Fauver et al. identified 14 coding-complete segments and 26 partial viral sequences including new insect-specific viruses belonging to the family *Flaviviridae* and *Totiviridae* [17] in the west African *An. gambiae* mosquitoes. Pinto et al., have identified six novel viruses in 66 *Culicinae* mosquitoes belonging to the 16 *Culicinae* species in midwestern Brazil by applying metagenomics methods [18]. A number of novel viruses have also been discovered in European arthropod species such as ticks and mosquitoes. Nine novel tick associated viruses have been sequenced from the *Ixodes ricinus* ticks from northern Europe [19] and three novel strains of rhabdoviruses have been identified in the *Ochlerotatus* mosquito species [20].

A group of vectors that to our knowledge have not yet been metaviromically studied are *Culicoides* midges, which are small biting flies. Most members of the genus feed on the blood of warm-blooded animals [21]. The recent discovery of *Schmallenberg virus* (SBV) [22] and the emergence of multiple strains of bluetongue virus (BTV) [23] in Europe have highlighted the important role that *Culicoides* species play in the rapid spread of viruses of veterinary importance across Europe. Midges are also capable of transmitting viruses to humans, as in the case of Oropouche virus (OROV) in Brazil [24]. The virome of midges other than pathogenic arboviruses has to our knowledge not yet been described but may be relevant in assessing future risks associated with these insects. Here we described the viral diversity in Scottish *Culicoides* midge populations. There are at least 41 different species of *Culicoides* biting midge described in the UK, of which 37 are present in Scotland [25,26]. We used HTS to build a better understanding of the viral diversity in the widespread biting midge, *Culicoides impunctatus*. In Scotland, this midge species is a major source of nuisance biting to humans, and also a vector of avian malaria. We report a detailed metagenomic analysis of the viral communities within *C. impunctatus* midges including the identification of seven novel viruses.

## 2. Materials and Method

### 2.1. Sample Collection and Sequencing

Three haematophageous midge pools (*Culicoides* spp.), formed of 10 midges each were collected during July 2015 from two sites located within the Loch Lomond and Trossachs National Park, Scotland [27]. Two pools (M1 and M2) were collected in the oak woodlands immediately surrounding the Scottish Centre for Ecology and Natural Environment (SCENE) (56°07’34”N, 4°37’04”W). The third pool was collected at the edge of Cashel Forest near livestock and dispersed cottages (56°6’38.3”N, 4°34’37.2”W). The midges were morphologically identified as *Culicoides impunctatus,* the most abundant *Culicoides* species found in the area. The midge species was identified using an interactive identification key for *Culicoides* (Ceratopogonidae) females from the Western Palearctic region [28]. These midges were then crushed by pestle and RNA was isolated from the 10 midges using 1 ml Trizol followed by standard RNA extraction, including isopropanol precipitation, using glycogen as carrier. A TruSeq Illumina stranded library preparation was carried out with input of 250 ng RNA per sample, following the manufacturer’s protocol. Briefly, RNA was mildly fragmented followed by a reverse transcription step and double-strand DNA (dsDNA) was synthesised. This dsDNA was A-tailed, followed by adaptor ligation and amplification using 15 PCR cycles. Libraries were quantified, pooled and loaded on the Illumina MiSeq. Paired-end reads were generated with read length 2 × 150 bp. This yielded 2,132,297, 1,881,438 and 1,657,675 paired reads for midge pools M1, M2 and M3 respectively. The raw reads and assembled contigs sequences are submitted to European Nucleotide Archives with BioProject number PRJEB33833 and accession numbers LR701640–LR701660.

### 2.2. Metagenomic Analysis Using MetaViC Pipeline

**Figure 1:**
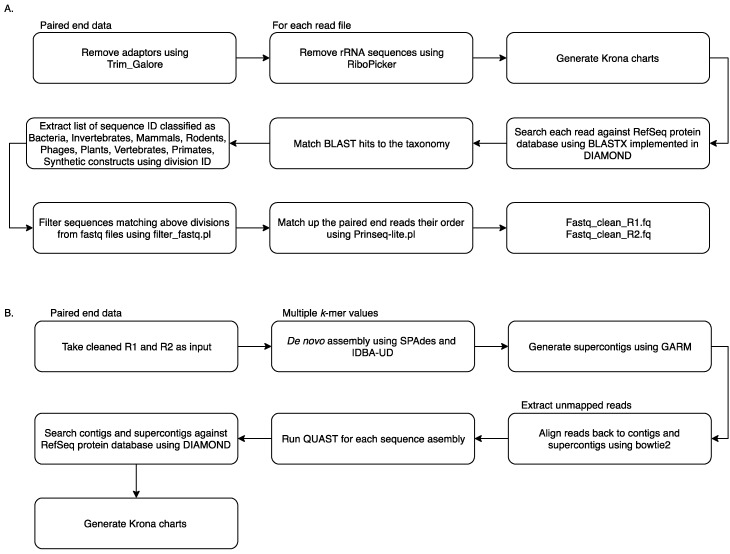
MetaViC workflow: (**A**) pre-processing step to remove unwanted sequences (**B**) de novo assembly and contig classification.

Reads from all samples were collated and analysed using the MetaViC pipeline, which has been designed to identify viral sequences in metagenomic datasets in the absence of a known host genome (the pipeline and documentation is available from: https://github.com/sejmodha/MetaViC) [29]. However, MetaViC can be applied directly to any virus metagenomics study. The MetaViC pipeline is divided into two major components (Figure 1). Firstly, reads were cleaned, and non-viral content was removed; secondly, the curated reads were assembled using de novo approaches. The reads were pre-processed by removing the Illumina sequence adapters using Trim_Galore [30]. Small and large subunits ribosomal RNA (rRNA) sequences were identified and removed using the in silico ribosomal sequence identification tool Ribopicker version 0.4.3 [31] with the SILVA rRNA database version 123 [32,33,34]. Each read was translated and searched against the RefSeq protein databases using a DIAMOND [35] blastx approach. The DIAMOND output was in turn parsed to identify and remove sequences that matched any known bacteria, invertebrates, mammal, rodent, phages, plants, vertebrates, primates and synthetic construct sequences. Read pairs that matched any known viral or environmental sequences and reads that did not match any known sequences in the RefSeq database [36] using an e-value of 0.0001 were extracted and kept for further analysis. At the end of this cleaning pipeline, sequences were confirmed to be properly paired using Prinseq-lite.pl [37]. These cleaned sequences were then submitted to the second component of the MetaViC pipeline that performed the de novo assembly.

The de novo assembly step was carried out using two *de bruijin* graph-based assemblers: SPAdes version 3.7.0 [38] and IDBA-UD version 1.1.2 [39] with multiple k-mer values ranging from 21 to 121. These two assemblers have been developed to reconstruct genomes with uneven coverage and depth which is typically the case for viral metagenomic samples [40]. The assembled contigs from the two tools were then consolidated using GARM version 0.7.5 [41], an assembly merging pipeline that uses MUMmer3 [42] to find overlaps between two assemblies and join them. The contig consolidation step is performed to generate supercontigs from both assemblies. This step is useful for constructing longer stretches of sequence and can also help to identify assembler-specific mis-assemblies and unique regions of the genomes from the shorter contigs generated independently by separate assembly tools [43,44]. Contig reconciliation also helps to improve assembly metric values such as N50. Reads were aligned back to these contigs and supercontigs using bowtie2 [45] and assembly statistics were generated using weeSAMv1.1 (https://github.com/centre-for-virus-research/weeSAM). Unmapped read pairs were extracted to retain any viral reads that might not have assembled into contigs. In order to check the assembly quality, QUAST [46] analysis was performed on each contig assembly and on supercontigs. Contigs longer than 200 nucleotides (nt) and supercontigs generated by GARM were then combined and classified using DIAMOND [35] against the RefSeq protein database. KronaTools [47] were used to create an interactive HTML output to visualise the formatted results generated by DIAMOND. Results obtained from the MetaViC pipeline were further investigated to identify viral signatures in the contigs. Any contig matching viruses that were run on the previous or same MiSeq run were excluded from the analysis (Table S1). 

### 2.3. Protein Domain Identification

The contig sequences that match viruses were translated using getorf [48] in all six frames keeping open reading frames (ORFs) with a minimum length of 300 nt. Local Interproscan [49] version 5.25–64.0 analysis was performed to search for protein domains present in the ORFs generated from the contigs. InterProScan domain search was applied to identify the known domain signatures within the ORFs. To put the newly identified sequences into the context of currently known viral genomes, each ORF sequence that contained viral signature domains was analysed using blastp [50] with e-value 0.001, and the top 20 hits were extracted. The top hits were used to produce a set of sequences for phylogenetic analysis. The metadata was extracted from the GenBank file for all sequences using a customised python script that collated details about protein accession, protein description, genome accession, RefSeq genome accessions, source, host and country of origin of the sequences. Multiple sequence alignments were performed using MAFFT [51] and converted to nucleotide alignments using pal2nal [52]. These alignments were used to compute the best substitution model based on the Bayesian Information Criteria (BIC) using jModelTest [53]. This substitution model was subsequently used for reconstructing a Maximum Likelihood phylogeny using RAxML [54] with node support evaluated using 1000 bootstraps replicates.

## 3. Results

### 3.1. Identification of Viruses from Midge Pools

**Figure 2:**
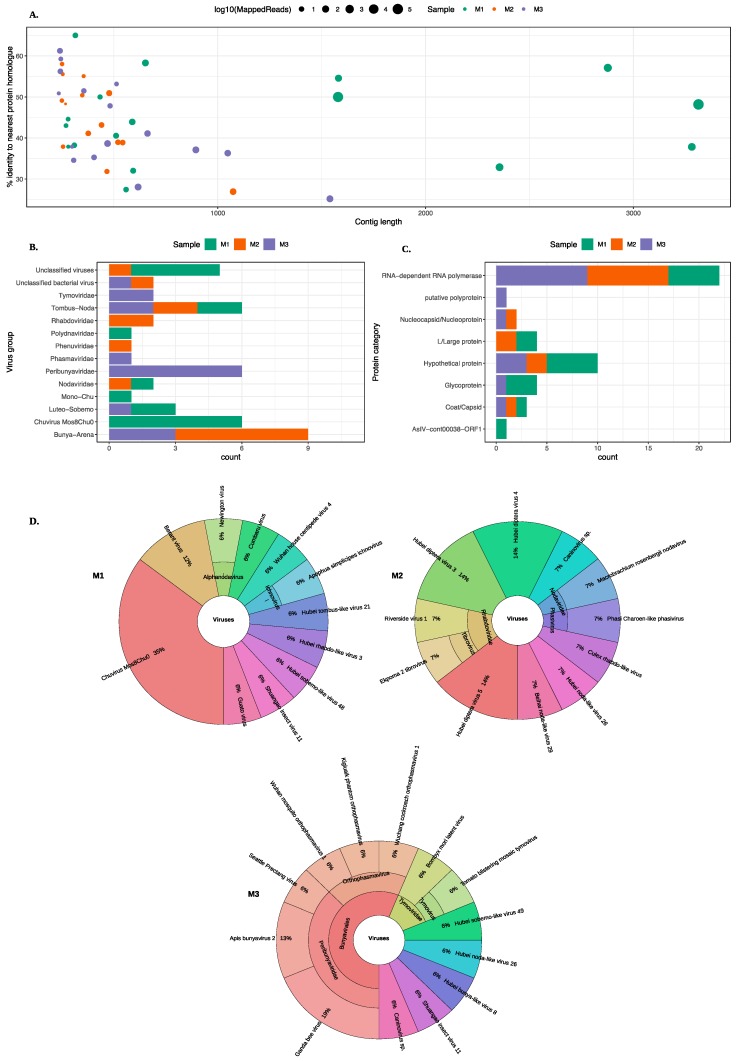
An overview of the virus hits found in all midge pools. (**A**) A scatter plot showing contigs lengths (x-axis) and the corresponding percent identity (y-axis) for the top BLAST hit. The colour of the circle represents the midge pools and the size of the circle represent the number of mapped reads on a log10 scale. (**B**) A bar chart describing different virus groups found in the midge pools where each pool is represented in a different colour. (**C**) A bar chart showing different proteins found in the midge pools characterised based on the nearest BLAST homologue. The virus classification groups and protein categories were derived based on the closest viral sequence match. (**D**) Distribution of the closest viral homologs found in the midge pools. Krona plots show the nearest virus hit found in all three midge pools, M1, M2 and M3 respectively. Colours in the Krona charts represent the distinct taxonomic groups.

MetaViC analysis found viral hits in all midge samples. Figure 2 and Table S2 summarise the DIAMOND results, showing the top hits for all contigs that matched viruses with a variable percent identity. As previously stated, the three midge pools are referred to as M1, M2, and M3 and contigs from each pool are referred to as MxCy.

An overview of the contigs matching viral protein sequences is shown in Figure 2. Most viral contigs identified in all three pools match with less than 60% protein sequence identity to known protein sequences. Most contigs were shorter than 1000 nt with exception of nine contigs (six in M1, one in M2 and two in M3) that were longer than 1000 nt. 

A range of different viruses were found in all three pools. Viral hits for these contigs spanned eight ICTV classified viral families, four unclassified groups and three unclassified categories of viruses. The viral hits to the group Tombas-Noda were common among all three midge pools whereas others including *Polydnaviridae*, *Phenuviridae*, *Phasmaviridae* and Mono-chu were exclusive to one of the sample pools. Further details about the closest virus homologues are shown in the Krona chart in Figure 2D. It is important to note that although the contigs from the viruses found in the *C. impunctatus* samples were classified as relatives of the viruses shown in Figure 2B, their sequences were significantly distinct compared to those that are currently present in the NCBI protein databases (Figure 2A).

The distribution of all protein hits is also shown in Figure 2C where the nearest BLAST protein homologs were grouped by the protein description. It is clear that most sequences match to RNA-dependent RNA polymerases (RdRp). The mean percent identity for these RdRp hits were around 44%. The highest percent identity was for M3C16 that matched RdRp of Wuchang Cockroach Virus 1 with 61% protein identity. The weakest and partial RdRp homolog was found in M3 with 28% protein identity to Seattle Prectang Virus. The second most abundant protein hits were to hypothetical proteins. These are usually the least studied sequences present in the databases with unknown functions. Other protein hits include nucleocapsid/nucleoproteins, capsid or coat proteins, and L/large proteins. Although these virus proteins are collapsed down to their functional category in Figure 2C, their viral origin varied among these contigs and more information about the viruses that these proteins originate from is available in the Table S2.

The most abundant hits were found for Chuvirus Mos0Chu8, as six contigs from M1 were found to be related to this virus. These proteins included RdRp, glycoprotein and hypothetical protein with varying sequence similarity from 32–58%. In pool M2, six different contigs match to the RdRp proteins from a range of Hubei diptera viruses with sequence similarity between 30–45%. 

Seventeen contigs from M1, 14 contigs from M2 and 16 contigs from M3 found hits to viruses in the NCBI non-redundant protein database. In this analysis, ORFs longer than 100 amino acids were investigated further using the InterProScan analysis. As most contigs had low sequence similarity to any known sequences available in the nr databases, a domain signature analysis was carried out. The domain-based analysis would help to identify the sequences that are more likely to code for the proteins.

For M1, a total of 28 ORFs were generated and 12 ORFs were identified to contain domain signatures from a variety of databases available within the InterProScan. For M2, ORF prediction led to the identification of 11 ORFs. Two of these 11 ORFs were found to contain protein domain signatures. For M3, five ORFs were shown to contain protein domain signatures out of a total 17 ORFs generated at the ORF prediction step. In summary, 19 ORFs combined from all pools were processed further in the downstream analysis. A phylogenetic analysis was performed for all ORFs larger than 100 amino acids generated from contigs that were larger than 500 nucleotides in size and matched a protein domain in InterProScan analysis.

### 3.2. Identification of a Novel Nodavirus from Two Midge Pools

**Figure 3:**
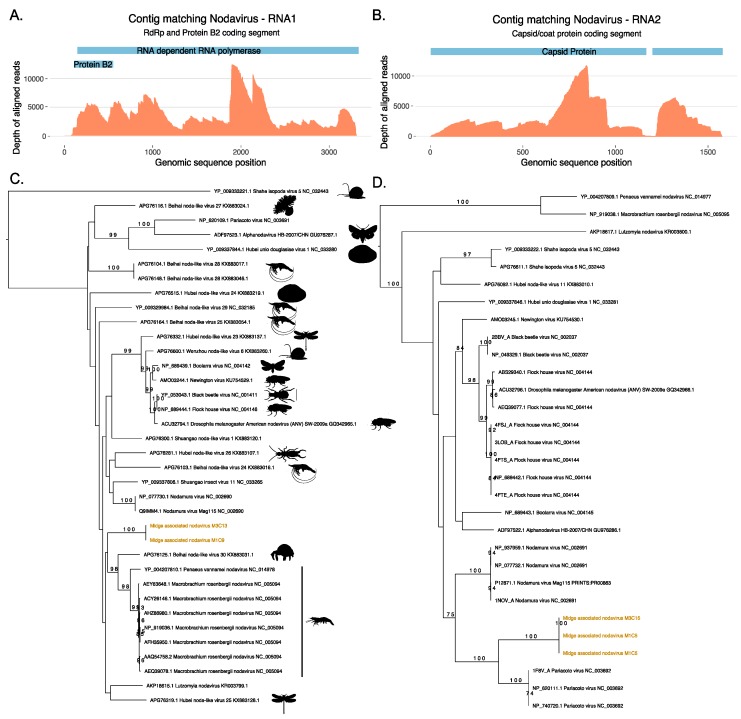
Novel nodavirus segments identified in the sample. Depth of coverage (orange) across the RNA1 (**A**) and RNA2 (**B**) segments with the coding regions for RNA dependent RNA polymerase (RdRp), Protein B2 and Capsid proteins shown in blue. (**C**) Phylogeny of the RdRp protein from the RNA1 segment and (**D**) phylogeny of the coat protein from the RNA2 generated using RAxML and showing the classification of the novel midge nodavirus RdRp and coat proteins with respect to existing protein sequences available in Genbank.

Two ORFs generated from the same contig M1C9 from M1 did not match protein sequences in the nr database, however, a longer ORF from the same contig was found to match a ssRNA virus, Shuangao insect virus 11 protein with low identity threshold, and a shorter ORF was found to contain RNA binding protein B2 signatures. Figure 3 shows the maximum likelihood tree for ORFs that match the coat protein of existing nodaviruses and their associated hosts. Nodaviruses are positive single stranded RNA viruses with two segments. The RNA1 is usually 3.1 kb long and codes for RdRp and RNA2 is 1.4 kb long and codes capsid protein [55]. The coat protein sequences from M1C5 and M3C15 matched the capsid protein of Pariacota virus with 56% and 36% amino acid identity, respectively. These ORFs, from M1 and M3, also contained Peptidase A6, nodavirus coat protein (Pfam ID: PF01829) signatures as described in Figure 3. Two separate ORFs from two samples, M1 and M3 were very similar along a region of 300 nucleotides shared between these two contigs with 100% similarity and clustered together with the capsid protein of Pariacoto viruses (Figure 3). This phylogenetic classification is well supported with a bootstrap value of a 100. Contig M1C5 comprised 63,523 reads, with a minimum depth of 111 across the entire contig. This evidence indicates the presence of nodavirus in the midge pools sequenced in this study. In these two midge pools, we also discovered two separate ORFs (M1C9 and M3C13) that matched RNA polymerase sequences of other nodaviruses. The top BLAST homologues of these ORFs were found to be similar to hypothetical protein of Shuangao insect virus 11, with 48% and 53% amino acid identity, respectively. The presence of the RdRp domain was confirmed with the InterProScan analysis for these ORFs. The domain signatures of the RNA polymerase proteins clustered under the SUPERFAMILY ID: SSF56672 and ProSiteProfiles ID: PS50507. We carried out a protein sequence alignment to find out the phylogenetic relatedness of these sequences with existing nodavirus RNA polymerases (Figure 3). However, bootstrap supports for the clade were very low and using a codon alignment for phylogenetic reconstruction did not improve the bootstrap support. Due to the low bootstrap support, it was not possible to determine whether the two segments are the result of a reassortment event but both segments are distinct to previously known viruses and their presence in two independent samples suggest that these ORFs originate from a novel nodavirus which is circulating in the midge population. As shown in Table S2, 148760 reads were mapped to midge associated nodavirus M1C9 with a complete contig coverage of 100% and average depth of 6183. The novel nodavirus identified in this study is called midge associated nodavirus followed by contig information. 

### 3.3. Identification of Rhabdo-Like Viruses in Midges

**Figure 4:**
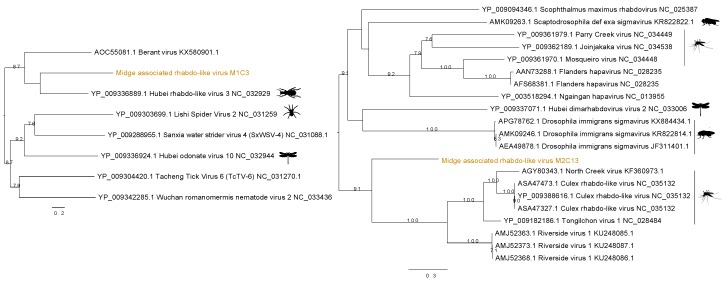
Phylogenetic tree showing the relationship of two novel midge rhabdo-like viruses identified in this study with currently known sequences from Genbank.

Two distinct sequences of rhabdo-like viruses were found in two separate midge pools. Figure 4 shows the contig M1C3 clustered with Hubei rhabdo-like virus 3 clade within the RdRp phylogeny with a bootstrap support of 59. This contig also contained an RdRp signature matching to RNA dependent RNA polymerase of the *Mononegavirales* in the Pfam databases with ID PF00946 and shared 37% amino acid identity with the RdRp of the Hubei rhabdo-like virus 3. Another contig from M2, M2C13, was found to contain the same RdRp signature from Pfam and shown to be clustered with *Culex* rhabdo-like viruses. Although M2C13 is distantly related to North Creek virus as seen by the long branches, it clusters with 91% bootstrap support with the clade comprising other mosquito viruses including North Creek virus and Riverside virus. The M2C13 ORF was shown to contain the *Mononegavirales* mRNA-capping region V signatures with Pfam ID PF14318. It showed 53% amino acid identity to putative RdRp of Tongilchon virus 1. Both phylogenies shown in Figure 4 were generated using the RdRp protein. The contig M1C3 clustered with Hubei rhabdo-like virus 3 that was found in the beetles (Coleoptera) whereas the M2C13 clustered with other unclassified members of the *Rhabdoviridae* family including Riverside viruses, North Creek virus, and *Culex* rhabdo-like viruses.

### 3.4. Identification of a Novel Chuvirus

**Figure 5:**
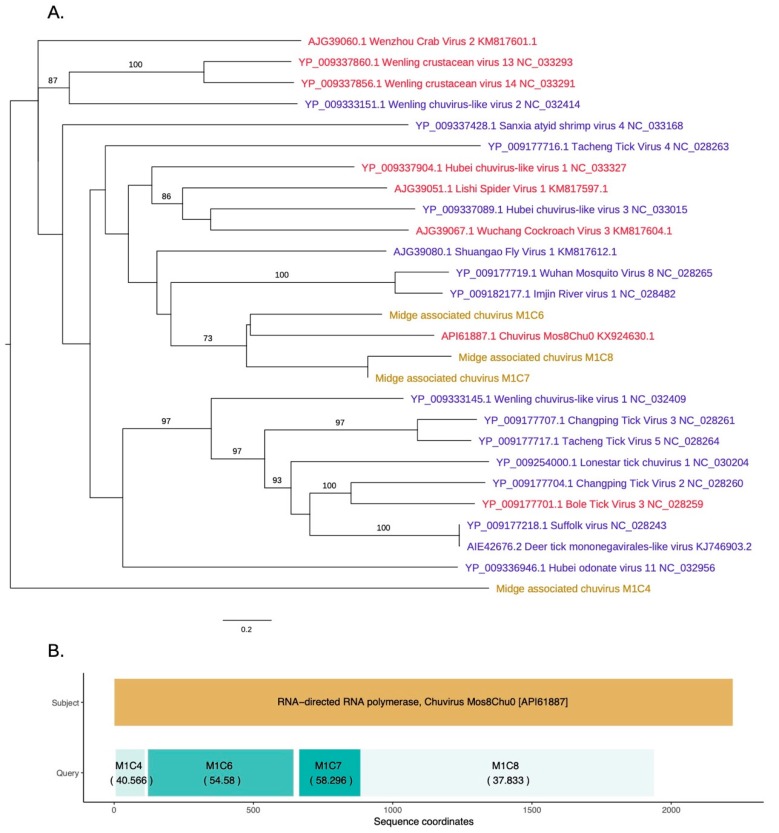
(**A**) RdRp based phylogeny describing clustering of a novel chuvirus species. Viruses that have two segments are highlighted with red tip labels. Blue tip labels highlight viruses that have one segment. Chuviruses found in the Scottish midge samples are highlighted in yellow. (**B**) Protein sequence similarity between Chuvirus Mos8Chu0 RdRp and midge chuvirus sequences with RdRp hits along with the sequence coordinates and coloured according to percentage identity.

As shown in Figure 5, novel chuvirus segments were also identified in the midge samples. Chuviruses are taxonomically classified under family *Chuviridae* and belong to the order *Jingchuvirales* (https://talk.ictvonline.org//taxonomy/p/taxonomy-history?taxnode_id=201856039). Three contigs M1C6, M1C7, and M1C8 were shown to be clustered with the chuviruses discovered in China. Another contig, M1C4, which has 40% identity to chuvirus RdRp, clustered with the recently sequenced chuviruses. These were non-overlapping contigs matching to the same RdRp sequences along the length of the subject sequence and may represent a single segment that is partially assembled due to insufficient data (Figure 5B). The contigs M1C6, M1C7, and M1C8 are in a clade with 60% bootstrap support that contains Shuango fly virus 1, Wuhan mosquito virus, Imjin river virus 1, and chuvirus. These viruses are found in hosts including *Culex* species and *Culiseta* species. Other hosts for chuviruses include crustaceans, ticks and dragonflies. The InterProScan analysis for the contigs M1C6 and M1C7 show the presence of the signatures of RdRp whereas the contig M1C8 contained the signature of mRNA-capping region V. The amino acid similarities, the branch lengths on the phylogeny and the pairwise distances among these three contigs (>75% at the nucleotide level) suggest that these three contigs are from three different parts of a midge chuvirus found in the same pooled midge sample. The phylogeny shown in Figure 5A is coloured according to the number of segments sequenced for each virus where the new sequences cluster with viruses that have two segments. Two other contigs found in the same pool, M1C2 and M1C15 have been identified to be similar to hypothetical protein and glycoprotein sequences of Chuvirus Mos8Chu0 with 30–40% protein similarity (Figure S1). However, InterProScan analysis did not identify any protein domains within these contigs. This is likely due to the lack of the captured diversity within this group of viruses. 

### 3.5. Identification of Bunya-Like Viruses

Novel bunya-like viruses were identified in the pool two and pool three, respectively. In M2, five different contigs were found to be similar to a range of bunyaviruses. Figure S2 illustrates the phylogenetic relationship of these contigs with respect to the currently known members of this virus group. This phylogeny is based on the RdRp sequences. One contig was closely related to Wuhan horsefly virus and the other four contigs were clustered in a lineage that contains Hubei diptera virus 3 and 4 that are members of the family *Phenuiviridae* in order *Bunyavirales*. It is important to note that the sequences identified within the M2 are likely to be partial sequences as these contigs are shorter than their phylogenetic relatives.

Three other phylogenies show the glycoprotein (Figures S3 and S4) and nucleoprotein (Figure S5) sequence-based classification for contigs that match bunya-like viruses with low protein sequence similarity. These contigs were found in M3 and clustered with Hubei odonate virus 8 and Seattle Prectang virus (*Peribunyaviridae*).

### 3.6. Identification of Luteo-Sobemo Like Viruses

Two separate ORFs that were found in contig M1C10 contained signature sequences matching hypothetical protein 1 and hypothetical protein 2, and clustered with the Hubei sobemo-like virus 48. The contig M1C10 shows 57% identity at the amino acid level to its closest BLAST hit and InterProScan analysis of these two ORFs identified signature sequences of peptidase S1 (SUPERFAMILY: SSF50494, Trypsin-like serine proteases) and Luteovirus RdRp (SUPERFAMILY: SSF56672, DNA/RNA polymerases). Figure S6 shows the maximum likelihood phylogenies for the two ORFs found in the contig M1C10. The phylogenetic bootstrap support was not very high for these ORFs as shown in the Figure S6. In both phylogenies based on two separate hypothetical proteins, the clade with ORF M1C10 is comprised of neighbouring viruses including Braid Burn virus, La Tardoire virus, Hubei Sobemo-like virus 48, Wuhan house centipede virus 5 and the Wuhan insect virus 34. Some hypothetical protein 2 sequences displayed in Figure S6B were annotated as RdRp-coding sequences on NCBI. Although the phylogenetic resolution was low, the low percent similarity at the amino acid level would indicate that we have found a novel species of Sobemo-like virus in the Scottish midge samples. Another contig from the same midge pool was clustered with a hypothetical protein of Motts Mill virus (Figure S7). The other viruses in the phylogeny have arthropod hosts such as *Drosophila*, millipedes and centipedes (*Myriapoda*), dragonflies (*Odonata*), spiders and beetles (*Coleoptera*) (Table S3).

## 4. Discussion

The emergence of SBV in Europe [22], which is transmitted by biting midges, and the incursion of multiple strains of bluetongue virus into Europe has driven a need to understand more about the diversity of viruses carried by biting midges. Moreover, recent data for mosquitoes has shown that even insect-specific viruses (unable to be transmitted to vertebrate hosts) can influence the vector competence of these mosquitoes for arboviruses, including human pathogenic ones [56], supporting the need for a deeper and broader understanding of insect-virus systems. In Scotland, *C. impunctatus* midge species is the major cause of nuisance biting on humans, causing a big drain on tourism. Some estimates suggest that 20% of forestry worker days are lost due to intense biting midge activities in Scotland [57]. *C. impunctatus* is also a vector of avian malaria [58]. Thus, we surveyed the viromes of this important species in two different locations in Scotland. We identified a range of viruses bearing very little similarity to previously known viruses with amino acid similarities ranging from 23% to 65% and thus the virome of the omnipresent biting midges in Scotland, is very distinct from any virome previously sequenced from arthropods. The differences observed between the two close locations suggests that the virome of *C. impunctatus* maybe geographically structured, and thus the virome of the biting midge is likely to expand further as midges are sampled over a greater geographical distribution.

In this study, we have found a range of nearly complete genomes of viruses from the families *Nodaviridae* and *Chuviridae* as well as partial genomes of novel viruses from the orders *Bunyavirales* and *Mononegavirales.* The limited availability of related sequences in the database resulted in poorly resolved phylogenies for some of the newly identified viral genomes. Whilst the position of coat protein of the novel midge nodavirus is very well defined and supported, we were unable to confidently assign RdRp coding segment to a particular clade due to poor bootstrap support. As more sequences become available for insect nodaviruses this may help to improve the support for the position of the RNA1 sequences. The host ranges of the nodaviruses include a number of insects and other arthropods such as butterflies, moths, *D. melanogaster*, *D. immigrans*, earwig, *Odonata* (dragonflies), hermit crab, and shrimps. A number of viruses have specific hosts such as channelled apple snail for Shahe isopoda virus 5 and Wenzhou noda-like virus 6 whereas other well studies nodaviruses including Black beetle virus, Pariacoto virus, *Macrobrachium rosenbergii* nodavirus have a wider host range and can be found in a number of invertebrates [55]. However, alphanodaviruses are not host specific and although nodaviruses are mainly isolated from insects, a member of the alphanodaviruses, *Nodamura virus*, also naturally infect pigs and herons and has the ability to kill mammalian and insect hosts [55]. Thus, it is unlikely that the midge associated nodavirus identified here is host specific. Additionally, it is clear from this study that the novel nodavirus is likely to be circulating in these midge population in Scotland as it was identified in two independent pools and its genome is identified and sequenced here for the first time.

A novel chuvirus found in the midge sample one is phylogenetically classified to be similar to the unclassified segmented virus Chuvirus Mos8Chu0 that was extracted from *Culiseta minnesotae*. This suggests that the chuvirus found in *C. impunctatus* is likely to be segmented. These viruses have recently been identified to belong to a newly generated virus family named *Chuviridae* that falls between the major groups of segmented and unsegmented negative-sense RNA viruses [4]. An example of such Chuvirus is a tick-borne virus, Jingmen tick virus, that contains genome segments derived from unsegmented viral ancestors. All three contigs from Scottish midge chuvirus contained RdRp signatures and were non-overlapping and spanned the complete segment of the same virus suggesting that we have identified a novel relative of the Chuvirus Mos8Chu0. In two other contigs we were unable to identify the expected glycoprotein domain suggesting that domain sequences and signatures within these species are unexplored and are still to be catalogued. Current evidence suggests that the diversity of these recently identified viruses is largely unknown and the phylogenetic relationship among this group of viruses would need to be updated as new relatives of these viruses are catalogued and their corresponding genomic diversity is captured.

Two distinct rhabdo-like viruses were found in two different *C. impunctatus* samples. Their classification as rhabdo-like viruses of the midges was well supported with strong bootstrap values. The rhabdo-like virus found in the midge sample two falls into a new branch with viruses such as North Creek virus. These viruses can be found in a range of mosquitoes, ticks, and midges including *Ochlerotatus sp.*, and *Culex sp.* (*Cx bitaeniorhynchus*, *Cx australicus*, *Cx globocoxitus*, *Cx quinquefasciatus*, and *Cx sitiens*). Other rhabdoviruses, such as Wuchan romanomermis nematode virus 2 included in the phylogeny can be found in the *Romanomermis* nematode. Rhabdoviruses have a wide host range and have been shown to switch hosts from mammals, fish, or arthropods [59]. However, it is unclear whether the midge rhabdo-like viruses might have arboviral properties such as the ability to infect vertebrates.

In this study, we have identified a large number of partial genome sequences from the *Bunyavirales* and *Mononegavirales*. This includes a novel relative of the Motts Mill virus and a relative of a segmented, unclassified member of the *Peribunyaviridae*, Seattle Prectang virus. The lower bootstrap and the higher diversity within these two orders of viruses indicate that these diverse viruses are relatively unsampled in these habitats and are yet to be discovered in this host range.

From this study and previous metagenomic studies of arthropods, it is evident that the currently known viral diversity from arthropod hosts is a small fraction of what must be evolving in the estimated seven million arthropod species [60]. However, with the lowering costs of sequencing and continuous improvements in sequencing technologies, viral discovery will accelerate. This will undoubtedly pose challenges to the taxonomic classification of viruses, which often needs expert knowledge and skills to submit classification proposals to the ICTV. Automated metagenomic pipelines such as MetaViC and virus sequence classification tools such as ViCTree [61] will help to streamline some of the steps involved in virus classification. In the current version of the MetaViC pipeline, a further sequence assembly tool such as digital normalisation could be added. The integration of such methodology within this analytical pipeline would add strength to the pipeline and may help improve overall assembly quality.

Though no viruses were cultivated, it is likely that these are naturally occurring viruses in the insect populations and not transcripts from genome incorporated viral sequences. The viral sequences identified in this study represent nearly complete viral genomes and genomic segments and contain structural, and non-structural protein signatures. This strongly suggest that the sequences identified in this study are highly likely to belong to novel; currently unidentified viruses and are captured and catalogued for the first time as part of this study. To our knowledge, this study represents the first virome from a midge species and it will be interesting to investigate whether the virome is shared with allopatric *C. impunctatus* populations and other sympatric *Culicoides* species to determine whether the viruses we have found are host specific or have geographically limited distributions. The metagenomic approach taken here could be scaled up and used as part of surveillance strategy to assess and describe potential future threats.

## Supplementary Materials

The following are available online at https://www.mdpi.com/1999-4915/11/9/865/s1. The following are added to this manuscript. Figure S1: Glycoprotein based phylogenetic tree showing the classification of the second segment of midge associated chuvirus found in M1. Figure S2: RdRp based phylogenetic classification of partial sequences of midge associated bunya-like viruses clustered with currently known bunyaviruses. Figure S3: Glycoprotein based classification of partial sequences found in midge pool three clustered with other bunyaviruses that occur in insects. Figure S4: A glycoprotein-based phylogeny for midge associated bunya-like virus M3C1 identified in midge pool three. Figure S5: Nucleocapsid based tree showing the phylogenetic relatedness of midge associated peribunya-like virus with other insect viruses in the group. Figure S6: Phylogenetic trees showing the classification of two ORFS from M1C10 for hypothetical protein 1 (A) and hypothetical protein 2 (B). Figure S7: A novel relative of Motts Mill virus identified using a partial sequence in the midge pool one. Table S1: Cross and between sequencing run contaminations found in midge samples, BLAST hits found to following viruses were excluded from further downstream analysis. Table S2: A comprehensive description of all contigs with a viral hit found in each midge pool with DIAMOND BLASTX homologs and the corresponding de novo assembly statistics. Table S3: Luteo-sobemo like viruses and their corresponding hosts.
