# Supplementary material for: Metaviromics Reveals Unknown Viral Diversity in the Biting Midge Culicoides impunctatus
Source: Viruses. 2019 Sep 17;11(9):865. doi: 10.3390/v11090865 (PMC6784199; doi:10.3390/v11090865)
Supplement: Supplementary file 1 [file viruses-11-00865-s001.zip › ManuscriptSupplementaryRevised/SupplementaryTables.docx]

**Table S1.** Cross and between sequencing run contaminations found in midge samples, BLAST hits found to following viruses were excluded from further downstream analysis.

| **Virus name** |
| --- |
| Bhanja virus (and related viruses including Heartland virus, Palma virus)  Langat virus  Uukuniemi virus (and related viruses including Grand Arbaud virus)  Canine mastadenovirus A  Cache Valley Virus  Kairi Virus |
|  |

**Table S3.** Luteo-sobemo like viruses and their corresponding hosts

| Virus name | Host |
| --- | --- |
| Braid Burn virus | Drosophila Subsilvestris |
| Hubei sobemo-like virus 43 | Arthropod |
| Hubei sobemo-like virus 48 | Coleoptera |
| Hubei diptera virus 14 | Diptera |
| Wuhan house centipede virus 5 | House Centipede |
| Wuhan insect virus 34, Shuangao sobemo-like virus 2, Shuangao sobemo-like virus 3, Wuhan insect virus 17 | Insects |
| Hubei sobemo-like virus 44 | Myriapoda |
| Hubei diptera virus 14, Hubei sobemo-like virus 42, Hubei sobemo-like virus 45, Hubei sobemo-like virus 49 | Odonata |
| Beihai sobemo-like virus 27 | Penaeid Shrimp |
| La Tardoire virus | Scaptodrosophila Deflexa |
| Wenzhou shrimp virus 9 | Shrimps |
| Hubei sobemo-like virus 40, Hubei sobemo-like virus 46, Hubei sobemo-like virus 47 | Spiders |

^3^These hosts were identified from NCBI GenBank virus sequence files representing the viruses shown in the table.
