# Supplementary figures and images for: Metaviromics Reveals Unknown Viral Diversity in the Biting Midge Culicoides impunctatus
Source: Viruses. 2019 Sep 17;11(9):865. doi: 10.3390/v11090865 (PMC6784199; doi:10.3390/v11090865)

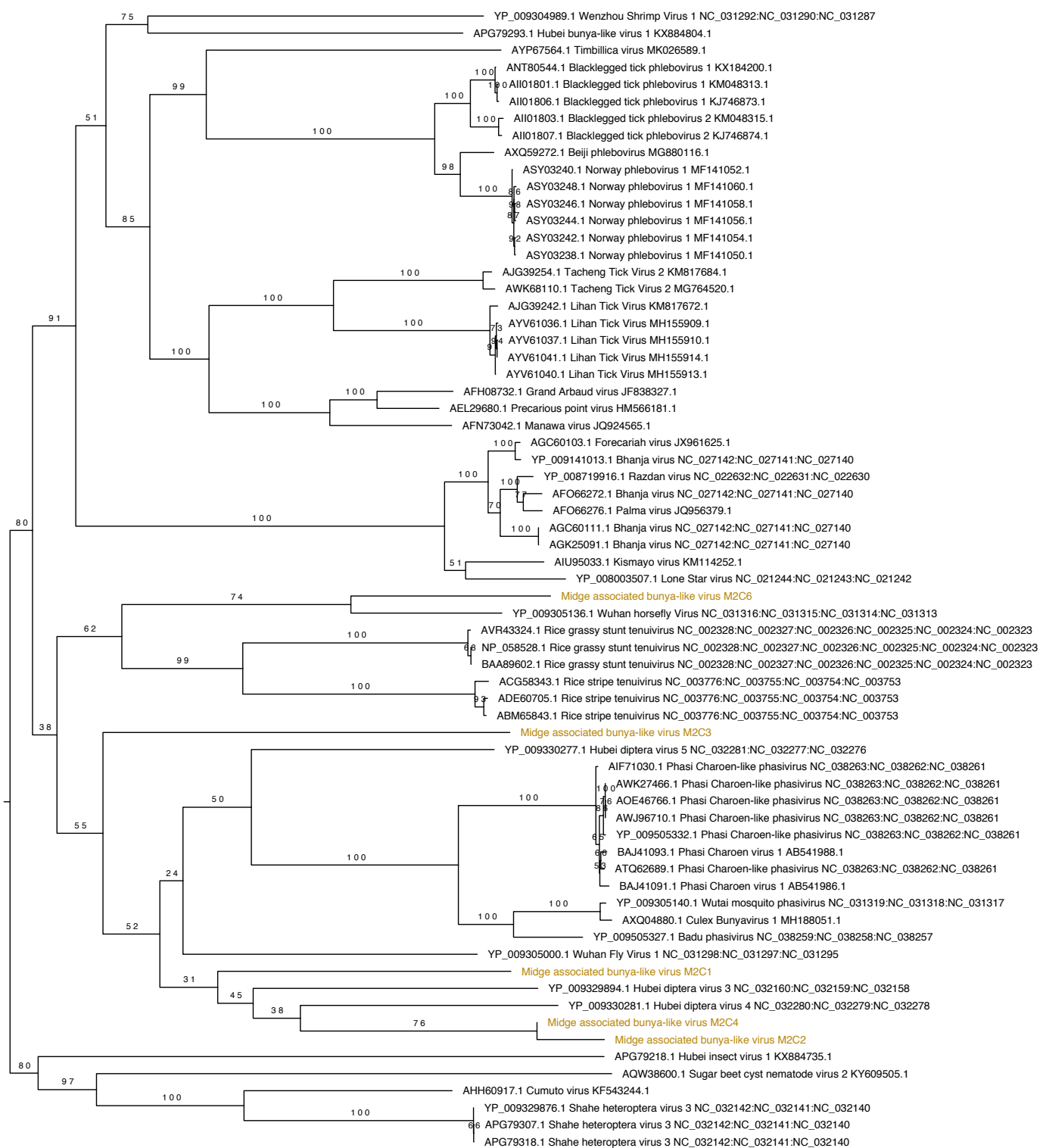

Supplement: Supplementary file 1 [file viruses-11-00865-s001.zip › ManuscriptSupplementaryRevised/FigureS2.pdf]

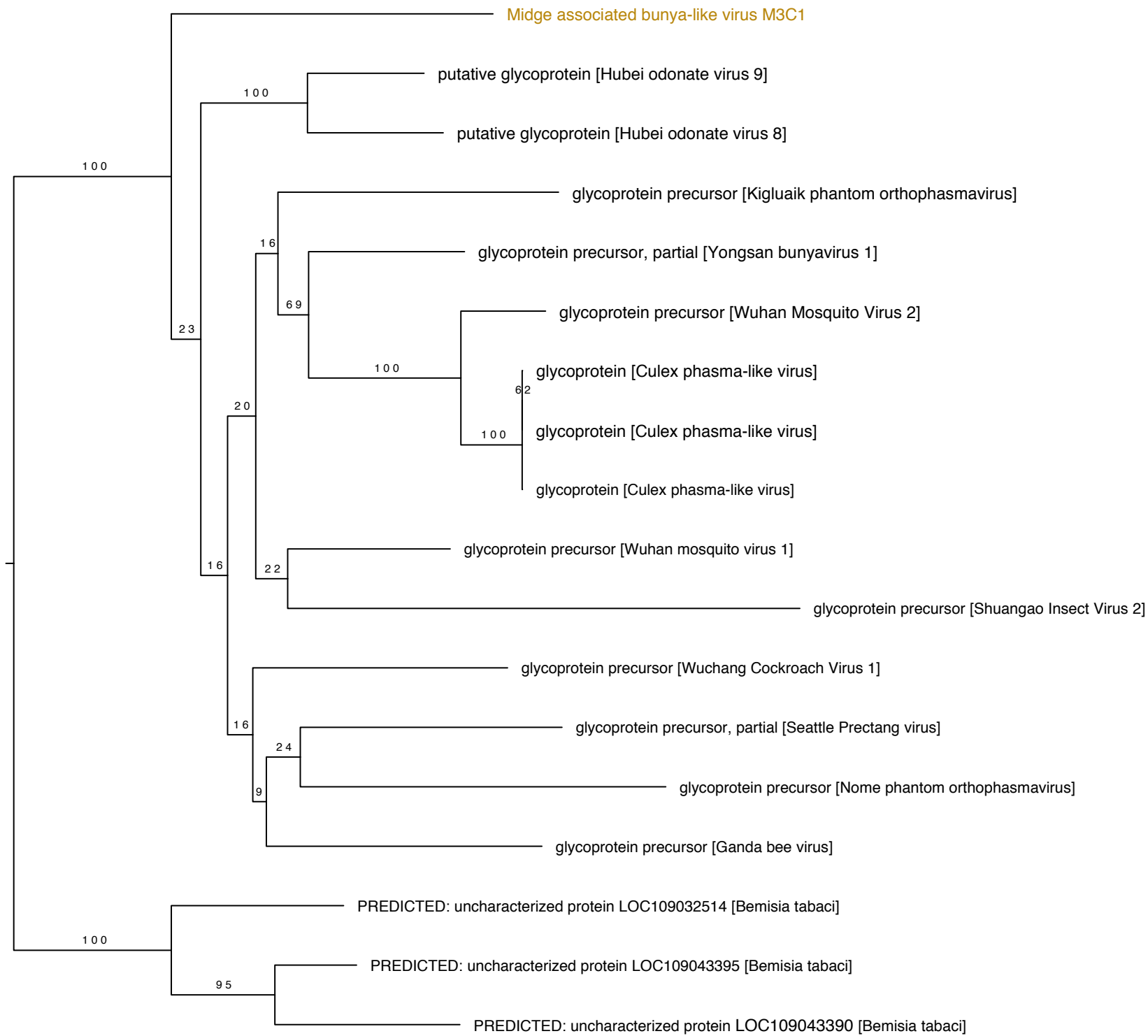

0.3

Supplement: Supplementary file 1 [file viruses-11-00865-s001.zip › ManuscriptSupplementaryRevised/FigureS3.pdf]

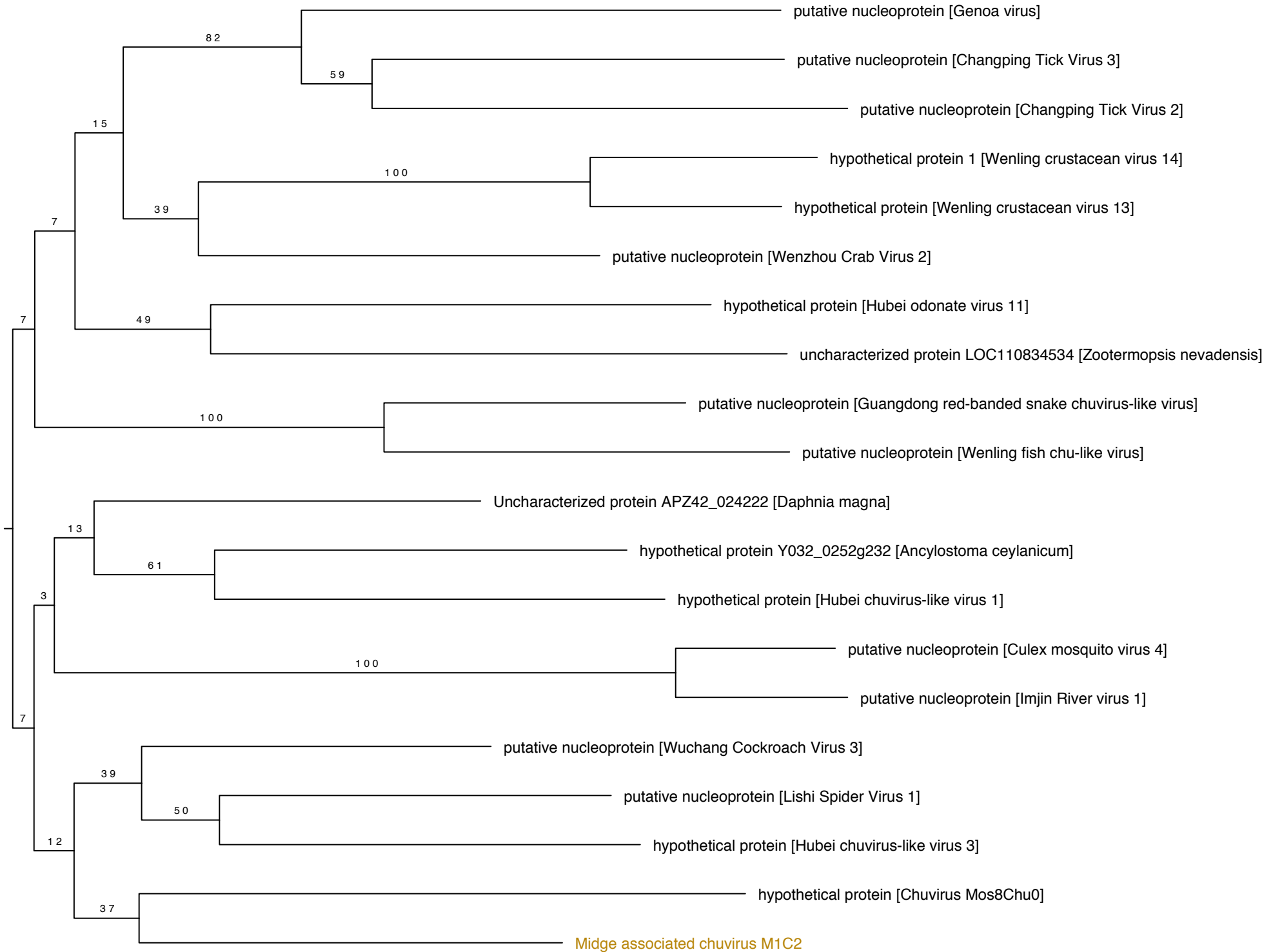

0.3

Supplement: Supplementary file 1 [file viruses-11-00865-s001.zip › ManuscriptSupplementaryRevised/FigureS1.pdf]

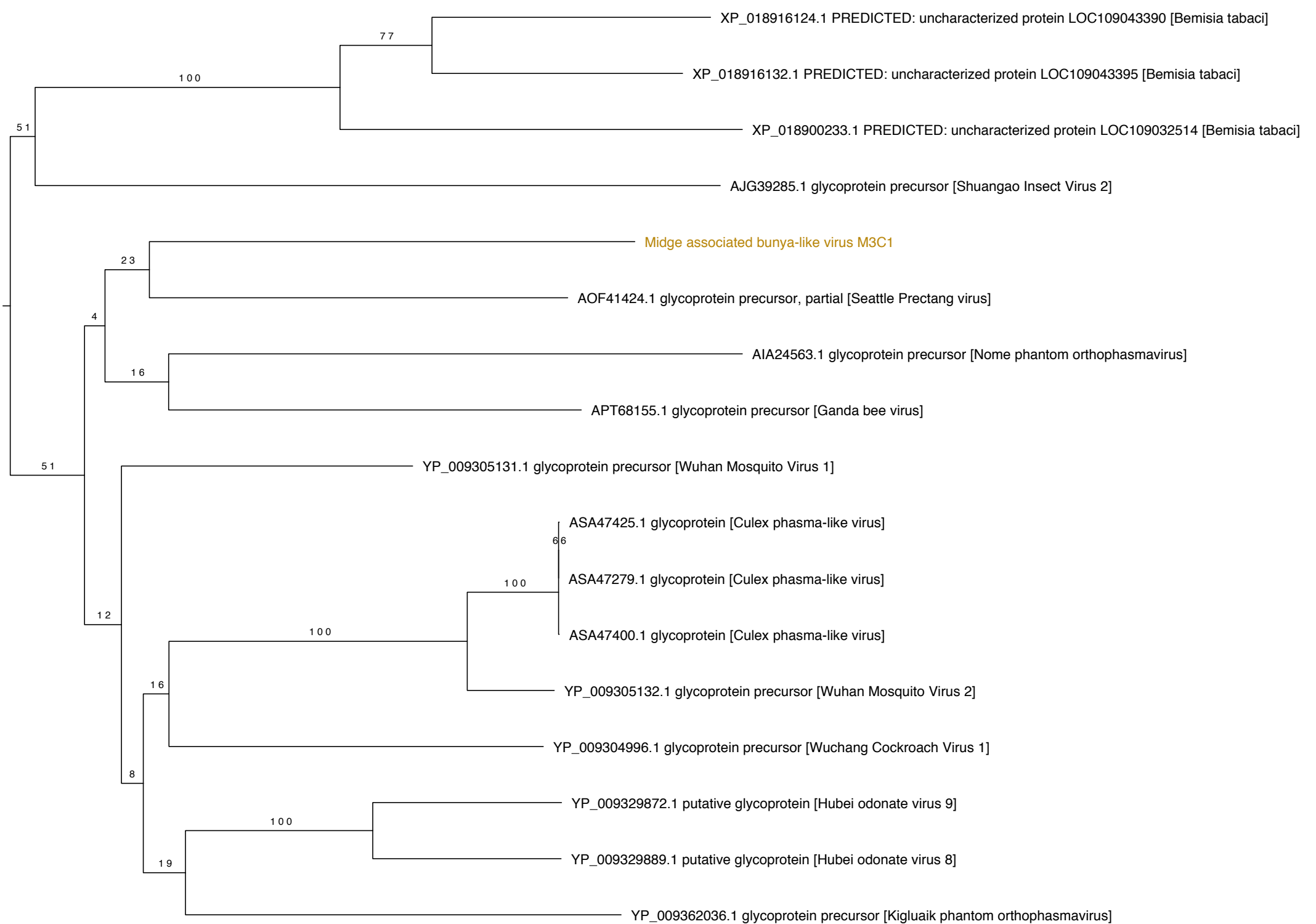

Supplement: Supplementary file 1 [file viruses-11-00865-s001.zip › ManuscriptSupplementaryRevised/FigureS4.pdf]

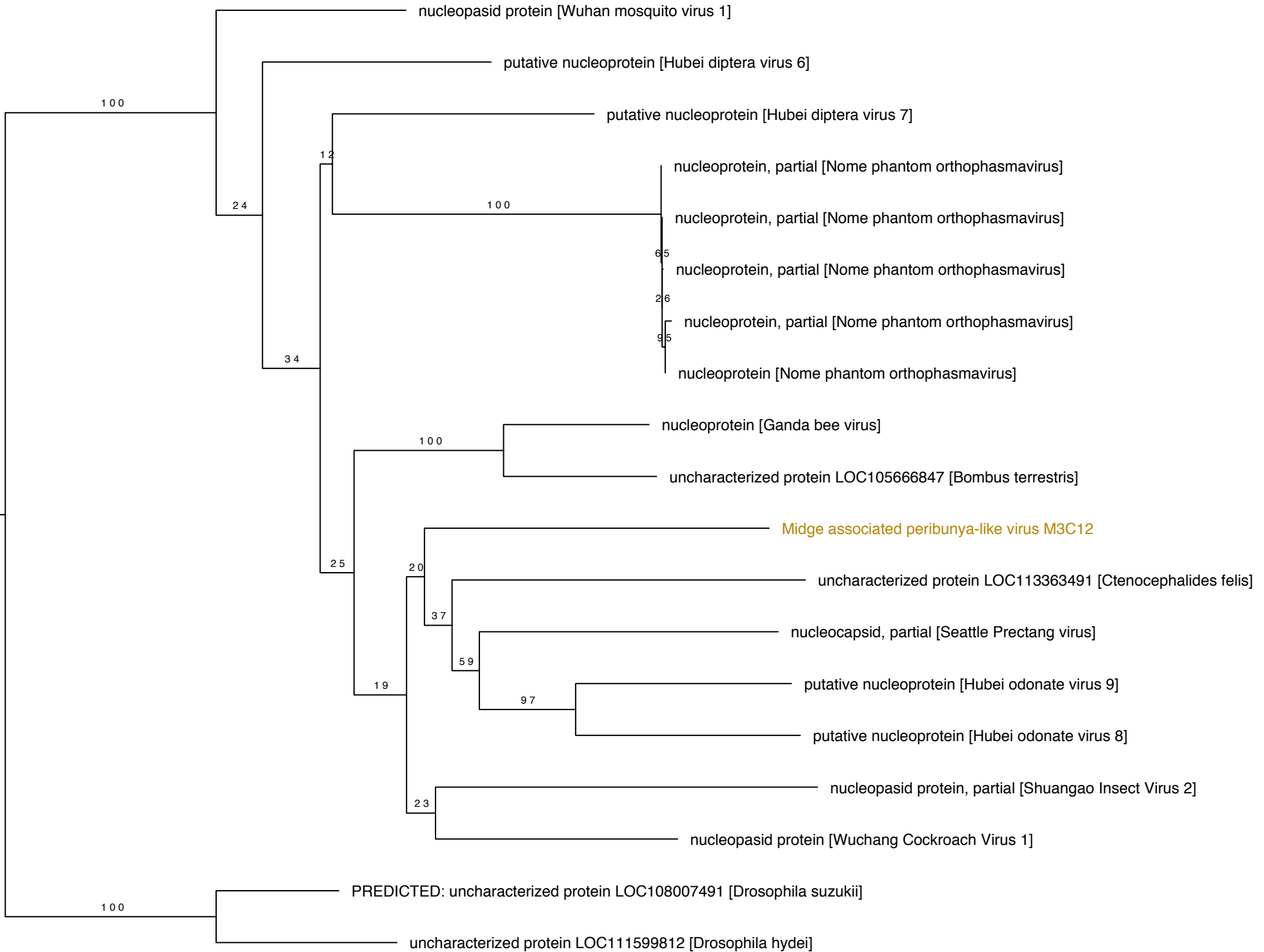

0.3

Supplement: Supplementary file 1 [file viruses-11-00865-s001.zip › ManuscriptSupplementaryRevised/FigureS5.pdf]

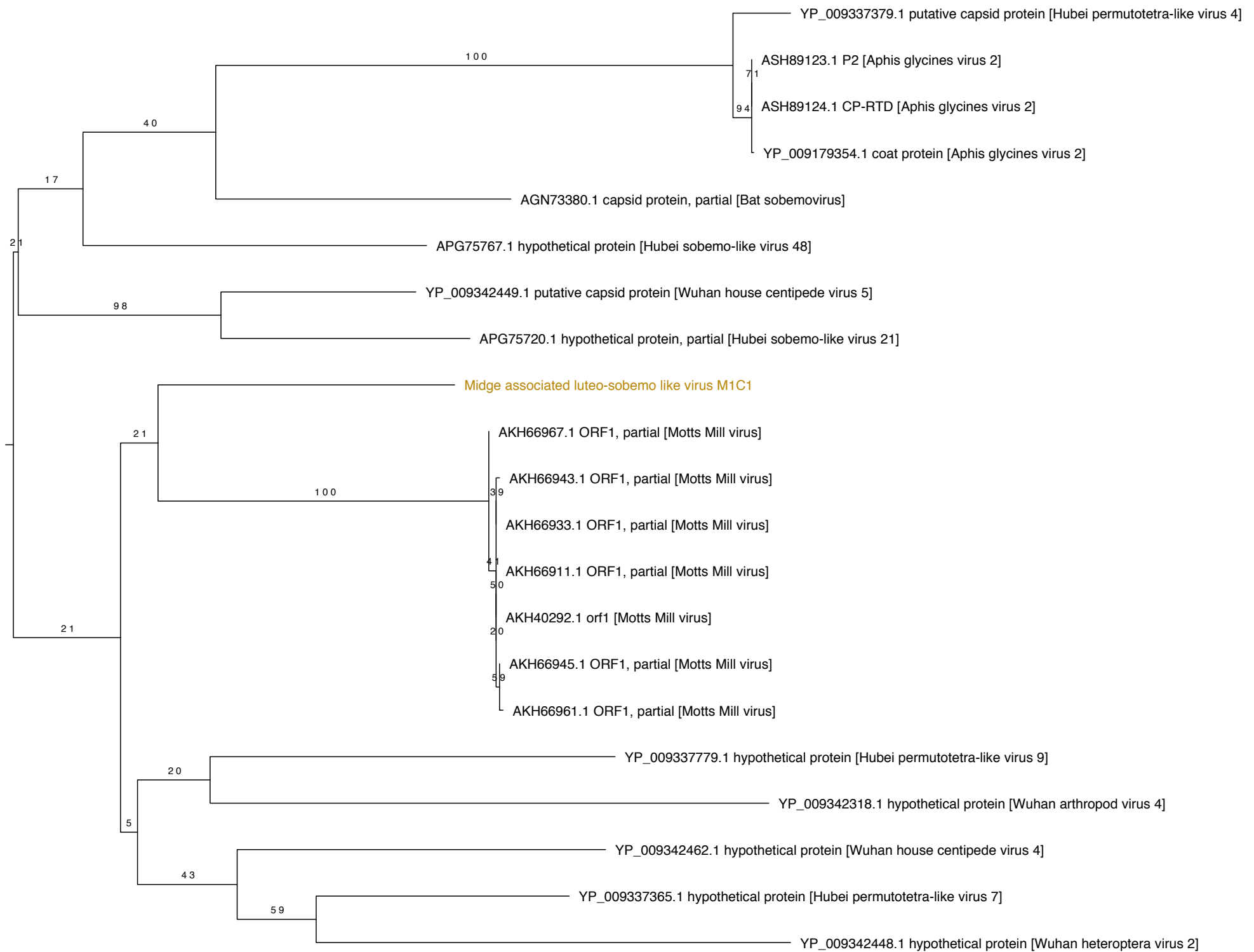

Supplement: Supplementary file 1 [file viruses-11-00865-s001.zip › ManuscriptSupplementaryRevised/FigureS7.pdf]

A.

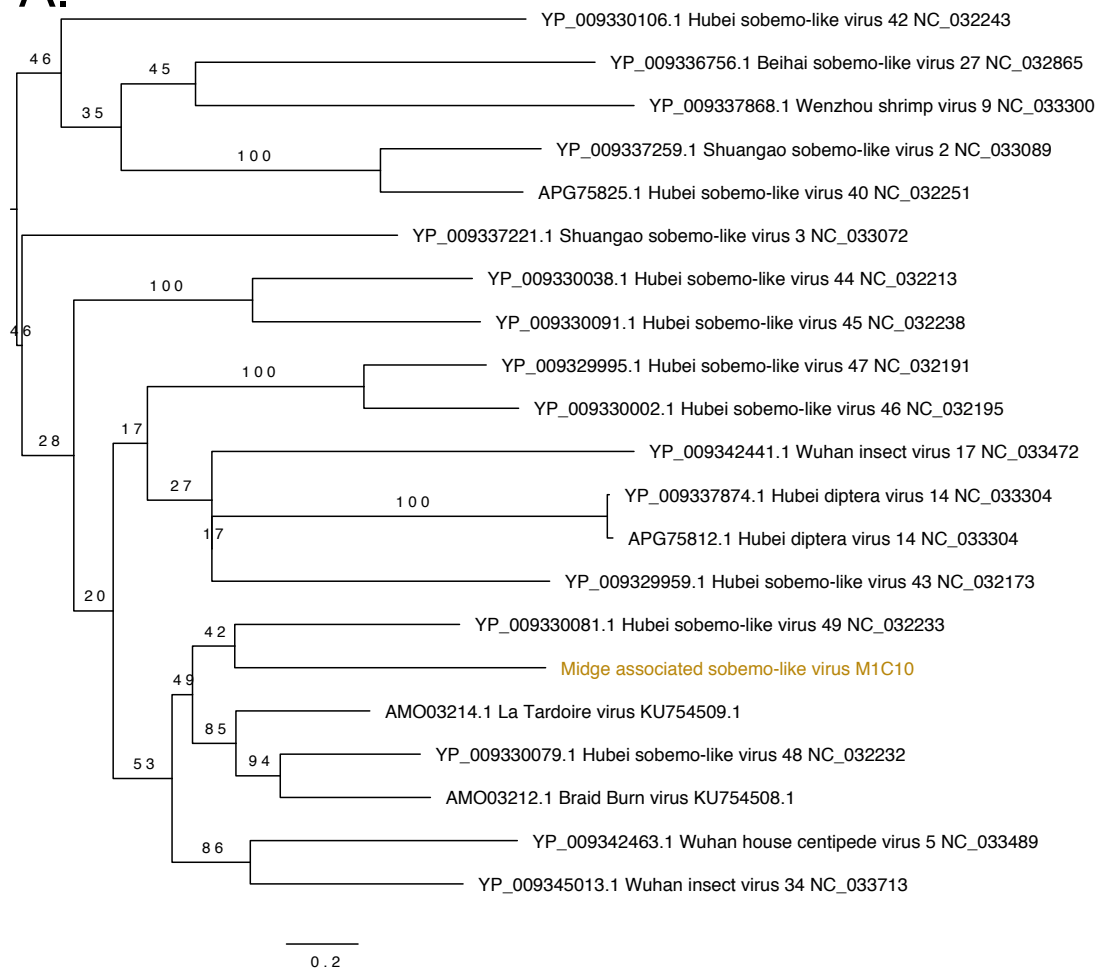

B.

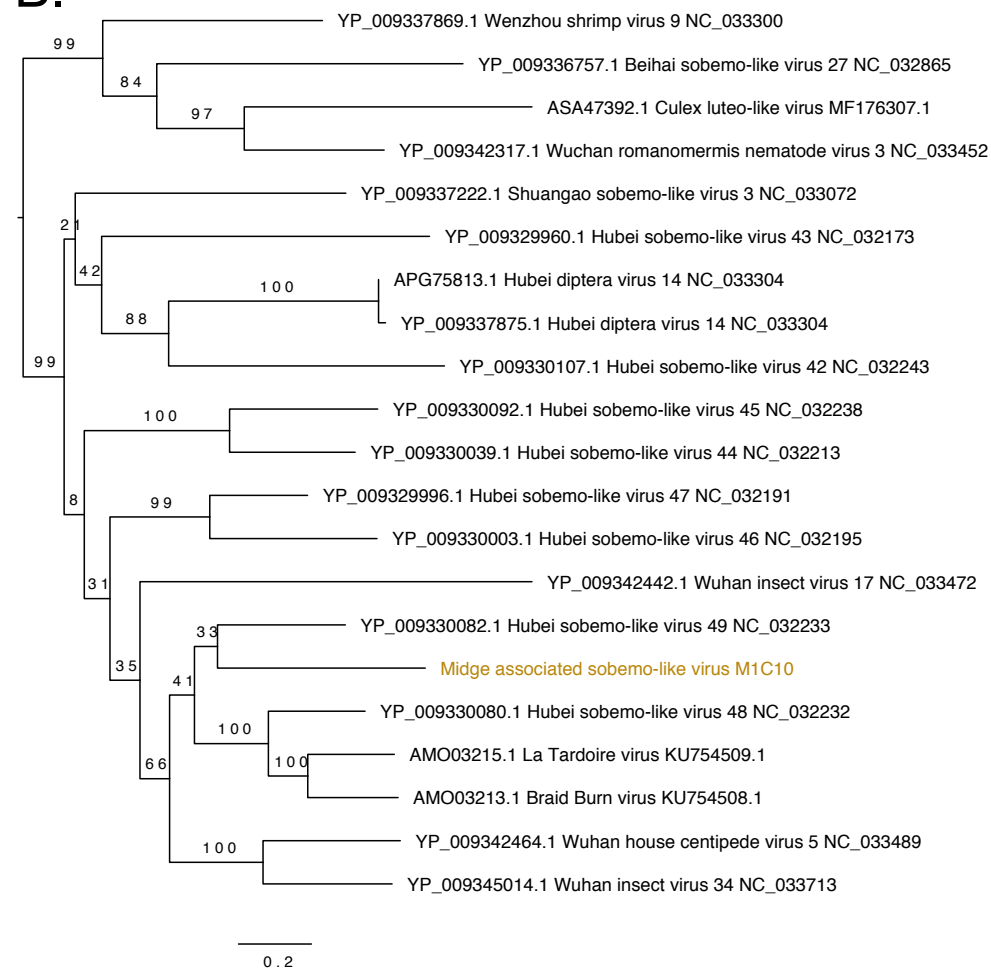

Supplement: Supplementary file 1 [file viruses-11-00865-s001.zip › ManuscriptSupplementaryRevised/FigureS6.pdf]
